# Why the disposable soma theory cannot explain why women live longer and why we age

**DOI:** 10.18632/aging.100253

**Published:** 2010-12-29

**Authors:** Mikhail V. Blagosklonny

**Affiliations:** Department of Cell Stress Biology, Roswell Park Cancer Institute, Buffalo, NY 14263, USA

The most influential line of reasoning in gerontology is known as Disposable Soma Theory (DST). In brief, the theory states that aging is caused by accumulation of random damage, which is counteracted by repair. Repair is costly and the organism allocates exactly the needed amount of energetic resources. Recently, DST was applied to explain why women live longer than men [1]. Women are less disposable than men, so they need a better repair and thus live longer [1].

It might seem slightly repetitive that women live longer because they are less disposable because females need better health for reproduction. I will discuss that this explanation is also erroneous. But to start with, the name of the theory (disposable soma) is ambiguous because soma is disposable by definition: soma *versus* germ line. All theories of aging are more or less disposable soma theories. (Footnote: according to DST itself, soma is not instantly disposable. It is constantly repaired depending on how much soma needs to be not be disposed of. Then soma is sort of recycled after constant repair). So we all agree that soma is disposable (by definition). The question is why is soma disposable and what makes it disposable. According to DST, it is allocation of resources from repair to other needs. Here I will also discuss an alternative model.

Let us consider that it is not lack of resources that renders soma unusable. According to TOR-centric model [2,3], as one of examples, there is another cause that kills us first. And this cause is not accumulation of random molecular damage. Yes, random damage accumulates with age. Yes, this eventually would make soma unusable. Eventually. But accumulation of random damage does not drive aging as we know it. Molecular damage does not cause *that* aging that kills us (and short-lived worms). *That* aging is an aimless, unintended, purposeless continuation of developmental growth and development is not driven by damage of course. While development is programmed, aging, however, is not. It is quasi-programmed (a quasi-program is an unintended continuation of a program that was not switched off after its completion [4,5]). And the same signaling pathways are involved in both development and aging. This is a clear-cut example of antagonistic pleiotropy. Activation of sensing-signaling pathways such as the nutrient-sensing TOR (Target of Rapamycin) drives growth and, when it is completed, TOR (among other players) causes aging. Figuratively, cellular aging is a continuation of growth [6]. Cellular aging (in part by causing age-related diseases) leads to non-random tissue, organ and system damage, ultimately causing organismal death. Driven by sensing-signaling pathways such as TOR, aging causes non-random macro-damage, literally visible in the mirror. In other words, diseases of aging and organismal aging results from chronic hyper-activation of intracellular signaling pathways such as the nutrient-sensing TOR pathway, which is antagonized by gerossuppressors or genes for longevity [5]. (on genes for longevity such as sirtuins, AMPK and FOXO see excellent reviews and commentaries [7-17]). (Footnote: Also, there are TOR-independent quasi-programs but more on this in forthcoming essays. Furthermore, activation of DNA-damage responses, even without damage, is a part (but not central) of TOR-centric network [18]). Cellular aging (hyper-activation) causes organ damage. This links gerontology to medicine, which deals with age-related diseases. Cellular hyper-activation (aging) via a chain of events - well known in medical science - culminates in non-random organ and systemic damage, for examples, damage of the heart and the brain (infarct and stroke). Once damaged, soma is not reusable any longer (unless medical interventions keep a patient alive). Thus, soma is instantly disposable after organ or systemic damage caused by cellular over-activation. Soma is like a car without brakes and without a driver too [19].

**So why do women live longer**? This was discussed in detail in the May issue of *Aging* [20]. In brief, high accidental death rate is associated with faster aging in different species, from worms to mammals. The same is applicable to longevity of males *versus* females. The accidental death rate, from accidents, violence, combat, is higher in young men than in women. Historically, it was much higher. Higher accidental death rate in young men may have led them to be larger and stronger than women. mTOR drives cellular size growth and muscle hypertrophy [21-23], including testosterone-induced hypertrophy [24,25]. (Noteworthy, rapamycin reversibly decreases levels of testosterone [26-28]). I suggest that hyper-active mTOR contributes to physical robustness of young males, allowing them to fight and compete. But hyper-active mTOR is beneficial earlier in life at the cost of accelerated aging. Thus males might age faster because TOR afforded strength and mass, which was beneficial in young males [20]. In other words, accelerated aging in males relative to females could be a byproduct of physical robustness to prevent death from extrinsic causes.Males need to be more robust and healthier to survive, outcompete other males and have a chance for reproduction. Females do not need such robustness (and health) to participate in reproduction and this is why they age slower and live longer. In contrast, DST postulates that females live longer because females need better health for reproduction.

Both TOR-centric model and DST agree that women live longer not because (or not only because) “men drive themselves to an early grave”. However, according TOR-centric model, exactly because “men drive themselves to an early grave”, evolution favored their robustness early in life and accelerated aging. Elimination of early death also explains “why longevity is constantly increasing”, by allowing slow-aging individuals to survive” [29].

But regardless of why women live longer, this mere fact seems to contradict DST. Females use more resources for reproduction and would be expected to have less for anti-aging repair. I will return to paradoxes of DST later.

Importantly, the TOR-centric model suggests means for extending health span: some are novel like rapamycin and some are well known like ‘eating less’. According to the TOR-centric model, food simply activates the nutrient-sensing TOR pathway, which in turn drives aging (or growth early in life). The more food, the more activated TOR, the faster aging [30].

Eating less, or calorie restriction, prolongs life span in most animals and prevents age-related diseases in humans, thus extending healthy life span. This cannot be easily reconciled with DST [31,32]. According to DST, anti-aging repair is costly and resources are scarce. More food intake corresponds to more calories *available* for repair. But more food intake, in contrast, corresponds to faster aging. To explain this observation, DST suggests that although repair is limited by scarce resources, the organism uses more resources for repair when resources are scarce, in order to delay aging (even though, according to DST, aging does not limit survival in the wild). Thus to explain why women live longer and why extra food shortens life span, classic DST was modified by suggesting paradoxical trade-offs and regulation of health depending on one's needs. Is trade-off between reproduction and somatic maintenance supported by data [33]. And does a pregnant woman halt DNA repair in her somatic cells? Other paradoxes were discussed in detail [32].

In its current form, DST implicitly contradicts evolutionary theory, suggesting that aging is regulated by choosing not to repair in the time of plenty, or instead repair when resources are scarce in order to live longer (as if aging limits lifespan in the wild) and reproduce later. According to DST, menopause is programmed to benefit both aging women and grandchildren. Although the grandmother hypothesis is extremely emotionally appealing (it provides positive meaning to menopause), women do not benefit from menopause. Simply most females did not live long enough in the past to experience menopause until recently. By its negative consequences, menopause is actually an age-related disease, like atherosclerosis, which occurs in every aging woman too. Like aging and atherosclerosis, menopause is not programmed and is not beneficial [20]. Like aging and atherosclerosis, menopause is quasi-programmed (an aimless continuation of reproductive program), an unintended by-product of development: the same mechanism that initially activates ovarian cycle later in life over-activates the ovary causing menopause unintentionally. This overactivation depends in part on the TOR pathway (see for ref. [20]). Menopause can be viewed as a by-product of development [34]. Loss of oocytes begins before birth and continues until menopause [35].

Proposed in the late 1970s, classic DST was not based on experiments but on pure logic. This is how an intelligent mathematician would design life: no waste; aging must be a passive process, whereas repair is limited by resources, depending on the needs of the organism (to be more or less dispensable). This is how life should be designed. But life was not designed by an intelligent scientist. It was shaped by a blind watchmaker for immediate benefits [36]. And it is wasteful and uses extra-calories to activate the TOR pathway even when it signals not growth, but aging.

Why is DST so popular? First, it is brilliantly presented, vividly written and published in most influential journals. As described in Scientific American “… aging process is caused by the gradual buildup of a huge number of individually tiny faults - some damage to a DNA strand here, a deranged protein molecule there, and so on”. Second, soma is disposable (according to all possible theories) so we all agree with the name of DST. But another reason for DST popularity seems to be that one needs no knowledge of biology or medicine to understand the theory. This is appealing. In contrast, understanding of TOR-centric theory requires knowledge of molecular mechanisms of signal transduction, clinical and experimental medicine and the genetics of model organisms. But there is a pay-off: We can make predictions and explain observations and suggest a therapy. Just by drawing signaling pathways from insulin and nutrients to mTOR with feedback inhibition of insulin-signaling, we can predict that low insulin levels (by not activating mTOR) extend life span (good health), whereas low insulin responsiveness can be a feedback response to over-activated mTOR, which shortens lifespan (bad health). In contrast, DST cannot explain this, thus creating paradox. According to DST, “The real paradox is why, in mammals, low insulin levels are associated with good health, but low insulin responsiveness with bad health” [37].

Thus, even though it requires background knowledge and efforts from the readers, scientific gerontology must be in agreement with medical science, genetics of longevity and molecular biology of signal transduction. It is studying of signal transduction pathways that can not only explain why women live longer but also how to extend lifespan without the need for a “drastic remedy” mentioned in the Scientific American paper [1].

**Appendix: not comparing apples to oranges.** One of the critiques of this essay was that “comparing DST and the TOR theory is like comparing apples to oranges. DST does not propose any molecular mechanism while the TOR pathway is a molecular network”. Although I was not intending to compare structures of the two theories, now I wish to add that both theories answer “Why and How” questions. According to DST “Why” is allocation of resources from anti-aging repair to other needs. “How” is accumulation of all sorts of random damage, with emphasis on molecular damage. (Footnote: The mechanism (How) might be different and, as an intellectual exercise, I can suggest another version of DST. For example, all tissues produce CO_2_ and energetic resources are needed to breath CO_2_out. But resources for breathing are limited. And one does not need to breath indefinitely; soma is disposable any way. This amusing version of DST - aging is caused by accumulation of CO_2_ due to limited resources- can seemingly make a strong case. For one, cessation of circulation or ventilation kills instantly. And *vice versa* ventilation of terminally ill patients extends life. Still, this is not a cause of aging). Having said all that, I would like to defend classic DST. In its original form, classic DST might be correct, and accumulation of random damage must terminate lifespan sooner or later. However, living beings die from another sort of aging first: non-random, quasi-programmed damage (mostly organ and systemic damage), manifested in humans as age-related diseases (in worm - other manifestations). The TOR theory, which is actually “quasi-programmed aging” theory also answers the questions Why and How. Why: Aimless continuation of genetic programs and processes, which are essential early in life but cause aging later. How: activation of signaling pathways that causes cellular aging and non-random organ damage such as myocardial infarction. To compare apples to apples, TOR-centric model should be viewed as quasi-programmed theory. And to compare oranges to oranges, DST is “life-long accumulation of random damage” theory. What is most important is not how to call a theory but how to suppress aging. And if aging is driven by processes that can be inhibited pharmacologically, then we may increase healthy lifespan in our lifetime [38].
